# Diffuse fibrosis in dilated cardiomyopathy results in a shorter myocardial null time

**DOI:** 10.1186/1532-429X-11-S1-P286

**Published:** 2009-01-28

**Authors:** Yuchi Han, Dana C Peters, Warren J Manning

**Affiliations:** BIDMC, Bston, MA USA

**Keywords:** Late Gadolinium Enhancement, Remote Myocardium, Diffuse Fibrosis, Diffuse Myocardial Fibrosis, Peak Signal Intensity

## Introduction

**Figure 1:**
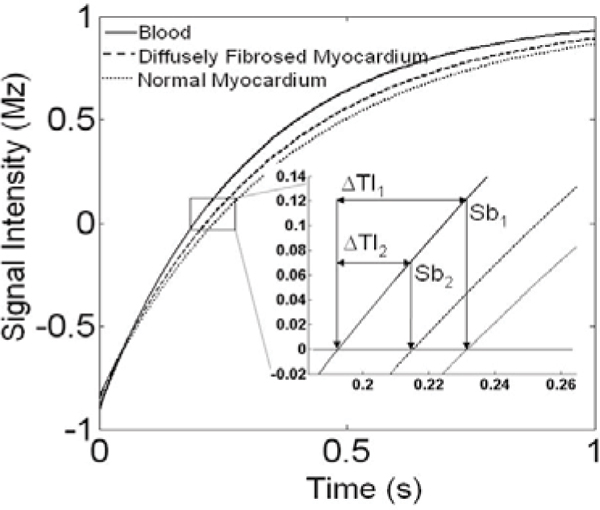
**Simulation of magnetization signal intensity regrowth curves of blood (T1 = 300 ms), diffuse fibrosis (T1 = 350 ms) and normal myocardium (T1 = 380 ms), after a 180° pulse**. Zoomed view shows the difference in null times between blood, diffusely fibrosed myocardium and normal myocardium (ΔTI_1_ and ΔTI_2_). Sb_1_ is the blood signal intensity when normal myocardium is nulled, and Sb_2_ is the blood signal intensity when diffusely fibrosed myocardium in nulled.

Biopsy studies have shown that 60% of dilated cardiomyopathy (DCM) patients have diffuse myocardial fibrosis. Late gadolinium enhancement cardiac MRI (LGE-CMR) has become an important tool in assessing myocardial fibrosis. Recently, studies have shown that LGE in DCM pertain a worse prognosis, including increased all-cause cardiac mortality. In these studies, LGE is considered present when the peak signal intensity is >2 standard deviations (SD) above the remote myocardium. However, in the presence of diffuse fibrosis, there is no remote "normal" myocardium, since the T1 of the entire myocardium is decreased. Figure 1 shows how diffuse fibrosis, with a shorter T1 than normal myocardium, will result in a shorter inversion time (TI) for nulling and a small difference in null points between blood and myocardium (delta-TI). Because of the shorter TI required to null diffusely fibrosed myocardium, blood signal in the LGE scan will be reduced. We sought to measure these effects in clinical scans of DCM patients.

## Methods

Ten DCM patients (age 64 ± 8 yr, 70% male) and ten heart-rate matched healthy control subjects (age 26 ± 9 yr, 30% male) were imaged on a 1.5 T Philips Achieva MR scanner (Philips HealthCare, Best, NL), equipped with a 5-element cardiac coil, using standard LGE protocol at 15–20 minutes post 0.2 mmol/kg Gd-DTPA (Magnevist, Berlex, USA) injection. Imaging parameters for the 2D LGE were: 2D spoiled gradient echo inversion recovery, 160 × 160 matrix, 320 cm FOV, 8 mm slices with 2 mm gaps, TR/TE/Flip angle = 4.3 ms/1.5 ms/20°, partial echo, fat saturation, 1 RR between inversions, 2 signal averages. A Look-Locker scan was used to determine the optimal TI performed at 10–15 minutes post-injection with a breath-hold 2D inversion recovery multi-gradient echo sequence with echo train length of 9 views, TR/flip angle = 40 ms/15°, 1 RR between inversions.

Region of interests (ROIs) were used to measure signal to noise ratio and contrast to noise ratio (SNR and CNR) on the short-axis mid-ventricular slice on the septal, anterior, inferior, and lateral walls, and in the blood pool. Noise was measured as the SD of signal in airspace anterior to the chest wall. The Look-Locker data were used to estimate the true zero-crossing for the blood and myocardial signal. ROIs were placed in the LV blood cavity and in the septal wall in each phase of the cardiac cycle. The zero-crossing for the signal was estimated using the linear interpolation between the two time points spanning zero signal intensity.

## Results

**Figure 2:**
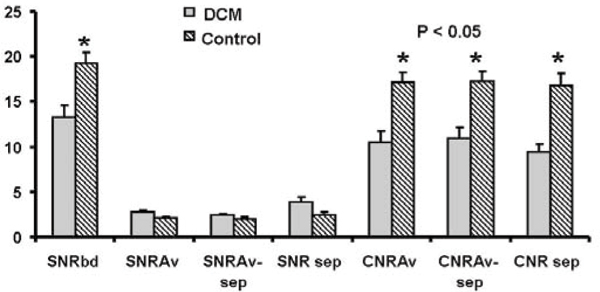
**SNR and CNR of the blood and myocardium**. SNRbd = SNR of the blood. SNRAv = Average SNR of the myocardium including the septum, anterior wall, lateral wall, and the inferior wall. SNRAv-sep is the average of SNR of the myocardium excluding the septum. SNR sep = SNR of the septal myocardium. CNRAv = Average CNR of the blood to myocardium including all 4 segments. CNRAv-sep = CNR of the blood to mhocardium excluding the septum. CNR sep = CNR of blood to septal myocardium.

**Figure 3:**
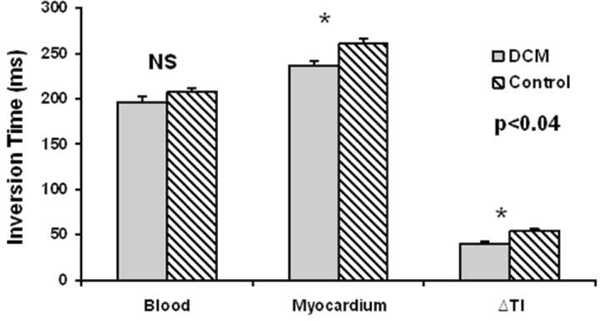
**Measured optimal inversion time (TI)**. DCM patients have lower optimal TI for myocardium and a smaller difference between the blood and myocardium TI (ΔTI) lower.

Blood SNR and blood to myocardium CNR were significantly lower in patients with DCM compared to controls (p < 0.05), but not the myocardium SNR (Figure 2). CNRs of the DCM patients were all significantly lower than the controls when averaging all walls, versus only the septum, or the rest of the walls without the septum (Figure 2). Figure 3 shows that the optimal TI to null myocardium was significantly shorter for the DCM patients when compared to controls (237 ± 24 ms vs. 261 ± 23 ms, p = 0.034). The delta-TI was significantly shorter (40 ± 10 ms vs. 54 ± 13 ms, p = 0.017) in DCM patients as compared to controls (Figure 3).

## Conclusion

DCM patients have significantly reduced blood SNR, a shorter optimal TI, and a shorter delta-TI, as predicted by the simulation in Figure 1. This provides evidence for the presence of diffuse fibrosis in patients with DCM. We did not find significant difference in the CNR of the septum vs. the remaining of the walls as reported by others using the remaining walls as remote myocardium. The detection of non-discrete fibrosis is a new aradigm for LGE-CMR.

